# Role of Dopamine β Hydroxylase (DBH) in Parkinson's disease patients of Indian population

**DOI:** 10.1186/1755-8166-7-S1-P122

**Published:** 2014-01-21

**Authors:** Arunibha Ghosh, Arindam Biswas, Tamal Sadhukhan, Shyamal Kumar Das, Jharna Ray

**Affiliations:** 1 S. N. Pradhan Centre for Neurosciences, University of Calcutta, Kolkata, India; 2 Burdwan Medical College & Hospital, Burdwan, India

## Background

Parkinson's disease is a neurodegenerative disease affecting at least 1% of the population over age of 55. It is characterized by selective loss of dopaminergic neurons in substantia nigra pars compacta and the appearance of intracellular inclusions termed Lewy bodies. As the age progresses, neurons in the other regions of brain are also degenerated. Depletion of brain dopamine initiates aberrant motor activities including rest tremor, rigidity, bradykinesia, postural instability. Apart from motor symptoms, cognitive impairments, like depression, psychiatric illness, and decreased mental ability are also observed in the patients. Genetic and non-genetic components are believed to govern the pathogenesis of PD. Genes in dopaminergic pathway & also dopamine synthesis, storage, binding, metabolism are needed to be studied which seem to be major determinants conferring differential risk of developing PD.

## Methods

In this study, three reported SNPs, rs1611115 (T>C), rs1108580 (A>G), rs129882 (C>T) of DBH have been analyzed. For rs1611115 (T>C), 229 patients & 252 controls have been screened. A total of 298 patients & 276 control samples have been analyzed for rs129882 (C>T). A total of 378 patients & 253 control samples have been analyzed for rs 1108580 (A>G). All these SNPs were screened by PCR , sequencing and/or RFLP analysis. Also, DBH activity has been measured in plasma isolated from patients’ blood.

## Results

For rs1611115 (T>C), the minor allele T is over represented in control samples & pose a protection (p=0.043, OR=0.731, 95% CI=0.731 (0.538-0.847) for this disease. No significant association has been found for rs1108580 (A>G). For rs129882 (C>T), the minor allele T is over represented in patient pool & confers a risk (p=0.036, OR=1.322, 95% CI=1.012-1.727) for this disease. DBH activity has been measured in plasma isolated from patients’ blood. A correlation has been found between plasma DBH activity & rs1611115.

## Conclusion

This study suggests that DBH might have a role in susceptibility of developing Parkinson’s disease.

